# Detailed characterizations of cranial nerve anatomy in E14.5 mouse embryos/fetuses and their use as reference for diagnosing subtle, but potentially lethal malformations in mutants

**DOI:** 10.3389/fcell.2022.1006620

**Published:** 2022-11-09

**Authors:** Lukas F. Reissig, Stefan H. Geyer, Viola Winkler, Ester Preineder, Fabrice Prin, Robert Wilson, Antonella Galli, Catherine Tudor, Jaqueline K. White, Timothy J. Mohun, Wolfgang J. Weninger

**Affiliations:** ^1^ Division of Anatomy, Center for Anatomy and Cell Biology, Medical University of Vienna, Vienna, Austria; ^2^ The Francis Crick Institute, London, United Kingdom; ^3^ Wellcome Trust Sanger Institute, Cambridge, United Kingdom

**Keywords:** phenotyping, cranial nerves, mouse embryo, reference data, HREM, topology, E14.5, diagnosis

## Abstract

Careful phenotype analysis of genetically altered mouse embryos/fetuses is vital for deciphering the function of pre- and perinatally lethal genes. Usually this involves comparing the anatomy of mutants with that of wild types of identical developmental stages. Detailed three dimensional information on regular cranial nerve (CN) anatomy of prenatal mice is very scarce. We therefore set out to provide such information to be used as reference data and selected mutants to demonstrate its potential for diagnosing CN abnormalities. Digital volume data of 152 wild type mice, harvested on embryonic day (E)14.5 and of 18 mutants of the *Col4a2*, *Arid1b*, *Rpgrip1l* and *Cc2d2a* null lines were examined. The volume data had been created with High Resolution Episcopic Microscopy (HREM) as part of the deciphering the mechanisms of developmental disorders (DMDD) program. Employing volume and surface models, oblique slicing and digital measuring tools, we provide highly detailed anatomic descriptions of the CNs and measurements of the diameter of selected segments. Specifics of the developmental stages of E14.5 mice and anatomic norm variations were acknowledged. Using the provided data as reference enabled us to objectively diagnose CN abnormalities, such as abnormal formation of CN3 (*Col4a2*), neuroma of the motor portion of CN5 (*Arid1b*), thinning of CN7 (*Rpgrip1l*) and abnormal topology of CN12 (*Cc2d2a*). Although, in a first glimpse perceived as unspectacular, defects of the motor CN5 or CN7, like enlargement or thinning can cause death of newborns, by hindering feeding. Furthermore, abnormal topology of CN12 was recently identified as a highly reliable marker for low penetrating, but potentially lethal defects of the central nervous system.

## Introduction

Biomedical model organisms are employed for researching the influence of genetic and biomechanical factors on embryogenesis and tissue remodeling. One of the most important models is the mouse. As a mammal, it has a basic body plan and genome comparable to humans. However, its pre- and postnatal life span and its generation times are much shorter (Waterston et al., 2002; Doyle et al., 2012). Combined with the availability of a broad variety of tools for altering the mouse genome (Bockamp et al., 2002; Rosenthal and Brown, 2007; Waerzeggers et al., 2010), this makes the mouse the most popular surrogate for researching the mechanisms steering human embryogenesis, hereditary diseases and malformations.

A high number of international projects build on the advantages of the mouse. Many simply alter the genome or the timely sequence of gene transcription and evaluate the effects these alterations have on the phenotype. Even large scale phenotyping programs, which systematically produce knockout (KO) mouse lines for every single gene were launched (Austin et al., 2004; Skarnes et al., 2011; Brown and Moore, 2012; de Angelis et al., 2015). Their first results were quite interesting, since they revealed that around one-third of gene knock outs produce lines, in which homozygous offspring die pre- or perinatally (Ayadi et al., 2012). They also revealed, that in 50% of those lines the embryos survive organogenesis, but die around or after embryonic day (E) 14.5. This triggered projects such as the Deciphering the Mechanisms of Developmental Disorders (DMDD) program (Mohun et al., 2013; Wilson et al., 2016), which aimed at systematically scoring the phenotype of embryos of KO-lines. DMDD in particular focused on phenotyping E14.5 embryos by employing digital volume data produced with the High Resolution Episcopic Microscopy (HREM) technique (Weninger et al., 2006, 2014). Despite the fact that at E14.5 organogenesis is largely completed and the individual is transitioning to the fetal period, the term ‘embryo’ is frequently use for this developmental stage (Brown and Moore, 2012; Adams et al., 2013; Mohun et al., 2013). Being aware of that, we also refer to E14.5 mice as embryos.

However, phenotype analysis of mouse embryos is not trivial. They face three serious obstacles:

First: Embryos harvested at identical time points and from the same dam often differ in their developmental progress. This dramatically reduces the validity of direct comparisons of the phenotype of mutants with that of genetically normal littermates and thus seriously hinders identifying abnormal phenotypes. To cope with this, staging systems were introduced. They categorize embryos by using similarities in characteristic morphologic features and thus rely on developmental progress rather than on age. Comparing two individuals of the same developmental stage then produces largely reliable results. The traditional staging system for mice is the Theiler system (Theiler, 1972). E14.5 mice are either of Theiler stage (TS) 22 or TS23. However, the anatomy of individuals of these Theiler stages still differs significantly. Consequently, Theiler staging recently was expanded and refined and a more precise staging system distinguishing six sub stages for mice of TS22 and TS23 was introduced by Geyer et al. (Geyer et al., 2017c)

Second: Gene knockouts do not only result in the absence of organs or gross abnormalities of their anatomical units. Instead, they often cause very subtle, yet functionally and clinically highly important structural defects (De Franco et al., 2019; Reissig et al., 2019). In particular, the diagnosis of abnormal tissue composition and architecture and deviations in the arrangement of three-dimensionally (3D) complexly arranged anatomical structures is challenging. Cutting edge imaging methods, producing highly detailed, high quality 3D digital data volumes are required to make them visible (Norris et al., 2013; Wong et al., 2014). As the most promising method to produce such data, we have identified HREM. Employing sacrificed individuals, it produces inherently aligned digital images of near the quality of histological sections, which can be instantly stacked to virtual 3D data with numeric resolutions down to 1 × 1 × 1 µm^3^. All commercially available 3D software packages can then be used for diagnosing organ and tissue abnormalities at all relevant levels of resolution (Weninger et al., 2014).

Third: Similar to adults, embryos/fetuses show an immensely broad spectrum of anatomic variation in topology, morphology and volume of tissues and organs, respectively their components. Hence, identifying anatomic variations and bordering them from pathologies and artefacts is far from being trivial (Reissig et al., 2021a). The only reasonable solution to cope with this problem is to compare the phenotype of each mutant with the phenotypes of a sufficiently large number of genetically normal individuals of identic developmental progress. Producing such data, especially of small anatomic structures of complex topology is a major effort and a time- and cost-expensive endeavor.

This needs reference data for detailed phenotype analysis of mouse embryos/fetuses to be based on high resolution volume data of a significant number of exactly staged wild type individuals. For the outlaid problems, meaningful 3D reference data of complex organ systems are scarce. Most of the existing literature elucidates specific mechanisms of CN development in the context of defined gene defects, investigates different developmental stages, is based on 2D information or lacks topological context of the surrounding structures (Kaufman, 1992; Eng et al., 2001; McNeill et al., 2010; Higashiyama et al., 2016; Wendling et al., 2021). Thus, they are suboptimal for the use as reference data for systematic phenotype analysis of E14.5 mice. Currently such reference data do only exist for the system, which has the highest priority for survival: the cardiovascular system (Weninger et al., 2009; Geyer et al., 2017b, 2022). These data are based on a sufficiently large number (approximately 200 data sets) of embryo data produced with HREM in the scope of the DMDD program. However, there are also other organ systems that urgently need to be addressed. In particular, the nervous system, which is also highly crucial for pre- and postnatal survival.

The central nervous system of E14.5 mice has already gained its general arrangement and shape, although the formation of nuclei and tractus has only just started. Also, the components of the peripheral nervous system are grossly formed. However, obviously, the thickness of nerves of adults and embryos, and even between embryos harvested at the same time point differ dramatically. This seriously hinders diagnosis of abnormalities of the topology and diameters of spinal and especially cranial nerves is almost impossible on the basis of existing two-dimensional (2D) descriptions.

To address this lack of 3D reference data, the present study aims at providing detailed anatomic and metric descriptions of the cranial nerves of mice of Geyer stages 21–23. The potential and value of these data to serve as a guide and reference for diagnosing cranial nerve abnormalities in mutant and genetically engineered mice shall be demonstrated by using selected DMDD mutants.

## Materials and methods

We analyzed HREM data of a total of 152 wild type and 18 mutant E14.5 mice, bred on the C57BL/6N strain at the Wellcome Trust Sanger Institute in the scope of the DMDD program and its pilot and side projects. All specimens had been staged according to Geyer et al. (Geyer et al., 2017c). 27 of the wild types were of stage (S)21, 12 of S22-, 17 of S22, 30 of S22+, 32 of S23- and 34 of S23. The mutants were from the *Col4a2* (4 individuals), *Arid1b* (7 individuals), *Rpgrip1l* (3 individuals) and *Cc2d2a* (4 individuals) KO-lines and had been staged as S22, S23-, S22 and S23- respectively. The KO-lines were included to demonstrate the usefulness of the reference data and were randomly selected amongst all DMDD lines featuring cranial nerve abnormalities.

Details on method and genetics of mouse production are provided in respective publications (Skarnes et al., 2011; Brown and Moore, 2012; Mohun et al., 2013). HREM data generation had followed standard protocols (Mohun T. and Weninger J., 2012; Mohun T. J. and Weninger, W. J. 2012; Geyer et al., 2017a). In short: The specimens had been fixed in Bouin’s solution for at least 24 h and then washed in phosphate-buffered saline (PBS), before having been dehydrated in an increasing series of methanol concentrations (10% increments with an additional step of 95%; at least 1 h for each step). After dehydration they had been embedded in methacrylate resin (JB-4, Polysciences, Warrington, PA, United States) containing eosin (0.275/100 ml solution) and acridine orange (0.056/100 ml solution). The blocks had been allowed to polymerize at room temperature and were then baked at 90°C for one to 2 days before having been subjected to HREM volume data production. Using conventional HREM apparatuses (Indigo Scientific Ltd., Baldock, United Kingdom), series of 2,000 to 4,000 digital images had been created from each specimen. They had been stacked to produce volume data with isotropic voxels with 2.55–3 µm sidelength.

The generation of digital volume data was followed by standardized phenotype analyses in the scope of the DMDD program. This was done employing a protocol aiming at the structured morphological assessment of all organ systems. The exact protocol is provided in Weninger et al., 2014 (Weninger et al., 2014). All found abnormalities were annotated in the volume data and assigned a Mammalian Phenotype Ontology (MP) term (Smith and Eppig 2009).

The HREM DMDD data were analyzed with the software package Amira 6.4 (ThermoFisher Scientific, Waltham, Massachusetts, United States) and Osirix (Pixmeo SARL, Geneva, Switzerland) by screening stacks of original or virtually re-sectioned digital 2D images and by using volume and surface rendered 3D computer models. Surface models were generated on the basis of binary files produced by outlining the nerve circumferences in virtual axial and coronal sections.

**FIGURE 1:**
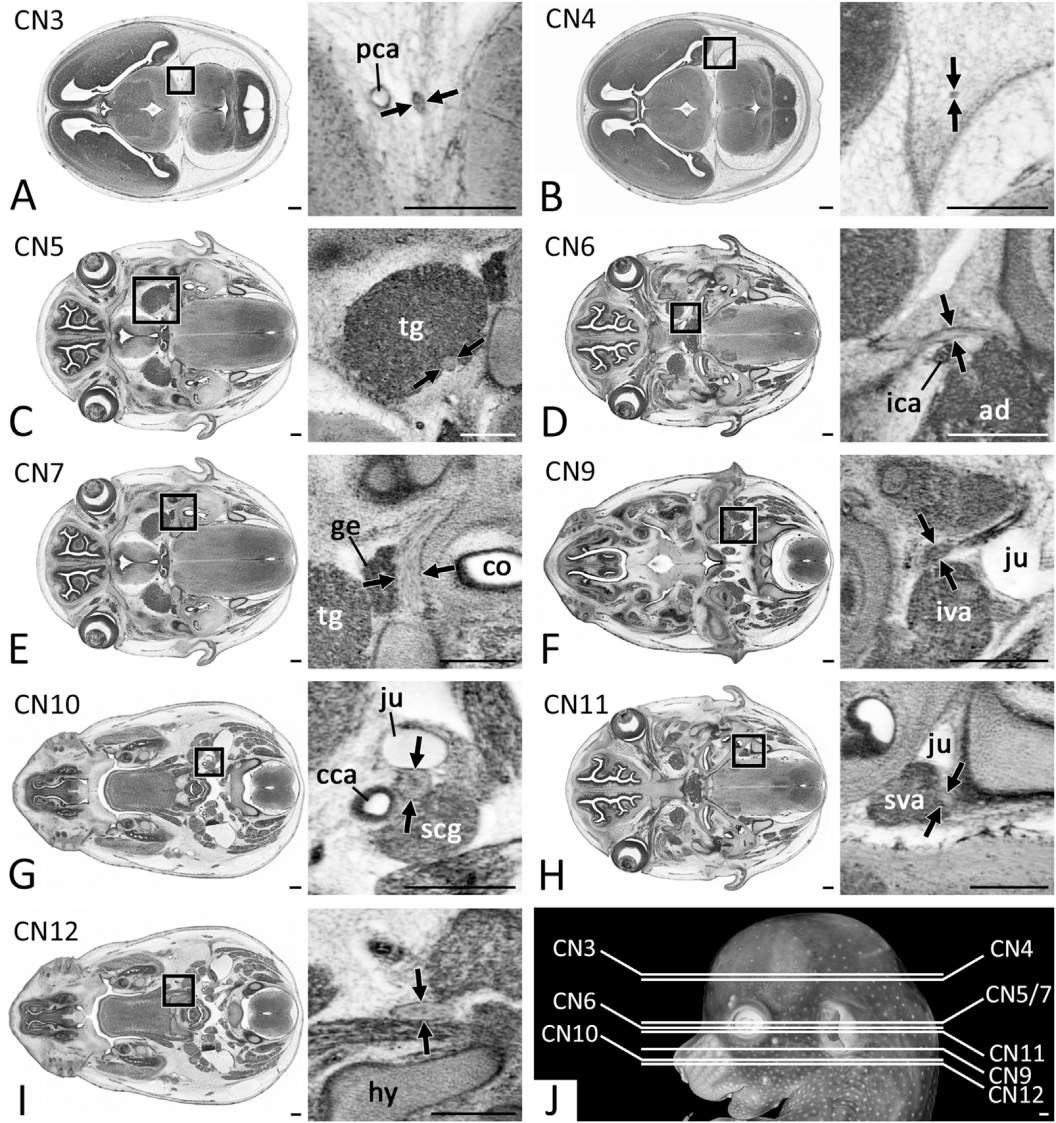
Positions of CN measurements (Arrows) in 2D axial HREM sections **(A)** CN3 measured after its rootlets form a solid nerve **(B)** CN4 measured within the forming dura mater **(C)** Motoric part of CN5 measured at its widest diameter within the trigeminal ganglion (tg) **(D)** CN6 measured next to the internal carotid artery (ica) **(E)** CN7 measured next to the geniculate ganglion (ge) **(F)** CN9 measured caudal to its inferior ganglion **(G)** CN10 measured caudal to its inferior ganglion **(H)** CN11 measured at its widest diameter next to the superior ganglion of the vagus nerve (sva) **(I)** CN12 measured lateral to the forming hyoid bone (hy) **(J)** Overview of the level of measurement positions. Abr. ad = adenohypophysis, cca = common carotid artery, co = cochlea, iva = inferior vagus ganglion, ju = jugular vein, scg = superior cervical ganglion. Scale bars = 250 µm.

The diameters of cranial nerves (CN) 3-7 and 9–12 were measured at defined and comparable positions (Figure 1). In order to produce results that can serve as references not only for volume, but also 2D section images and to keep the method simple and robust, the diameters were measured in carefully selected original, and thus axial 2D HREM section images using the 3D line measuring tool of the Amira^®^ software package. Nerve segments, which, in the images appeared as cut longitudinally were identified and subsequent HREM section images were screened unit the images, featuring the nerve in its maximal thickness was identified. Obliquely and vertically sectioned nerves were measured at their thinnest position. As CNs 1 and 8 were composed of multiple single nerve strands and CN2 semi-surrounded the optic stalk and recess, the diameters of those CNs were not measured.

Microsoft Excel (Microsoft Corporation, Albuquerque, NM, United States) and SPSS statistical software (IBM Corporation, Armonk, NY, United States) were used for descriptive statistics and statistical analysis. To assess the effect of the developmental stage on the CNs’ diameters we performed a one-way ANOVA followed by a Tukey post-hoc-analysis or a Welch ANOVA followed by a Games-Howell post-hoc-analysis depending on the homogeneity of variances. Homogeneity of variances was assessed using the Levene-Test, normal distribution of the data was assessed using the Shapiro-Wilk-Test. A *p*-value of ≤0.05 was considered statistically significant.

## Results

**FIGURE 2:**
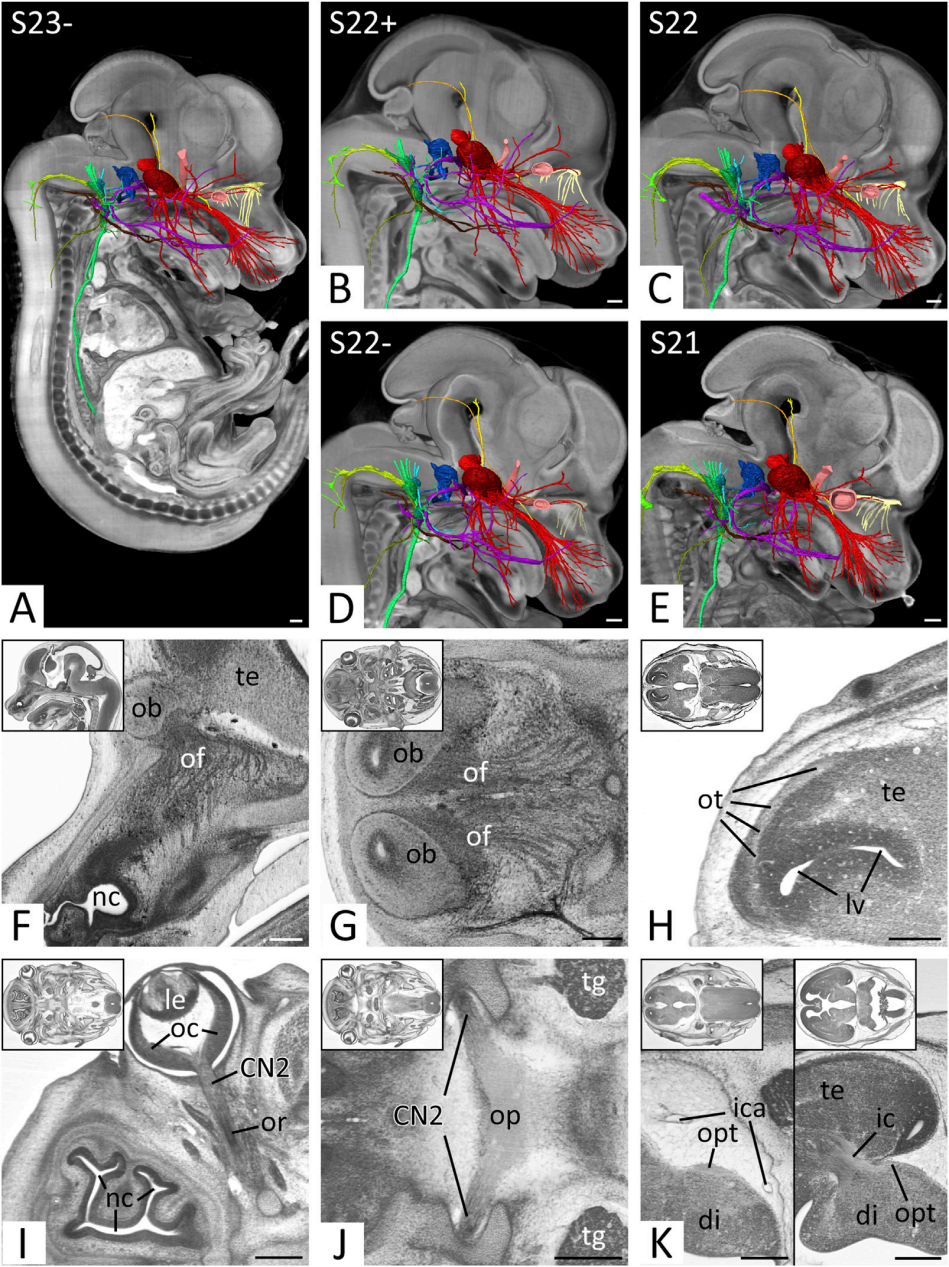
Overview and cranial nerves (CN) 1 and 2 **(A–E)** Overview of all right CNs. 3D surface renderings in the context of a volume rendering of a mouse embryo of S23- **(A)**, S22+ **(B)**, S22 **(C)**, S22- **(D)** and S21 **(E)**. View from right. The overview of S23 is shown in Figures 4, 5. CN1 (light yellow), CN2 (light red), CN3 (yellow), CN4 (orange), CN5 (red), CN6 (pink), CN7 (violet), CN8 (blue), CN9 (turquoise), CN10 (green), CN11 (olive), CN12 (brown) **(F–H)** Olfactory nerve (CN1) in sagittal **(F)** and axial **(G,H)** HREM resections. Anterior to the left, right on top. Inlays: Overview. **(F,G)** Olfactory fibers (of) from the nasal septum **(F)** and superior nasal turbinate **(G)** reaching the olfactory bulb (ob). **(H)** Olfactory tract (ot) on lateral aspect of the telencephalon (te) **(I–K)** Optic nerve (CN2) in axial HREM sections. Anterior to the left, right on top. Inlays: Overview. **(I)** Optic nerve (CN2) emerging from optic cup (oc). Note the optic recess (or) above the nervous tissue. **(J)** Optic plate (op) formed by both CN2s. **(K)** Course of the optic tract (opt) on the lateral aspect of the diencephalon (di) (left) to the occipital part of the internal capsule (ic) (right). Abr. ica = internal carotid artery, le = lens, lv = lateral ventricle, nc = nasal cavity, tg = trigeminal ganglion. Scale bars = 250 µm.

All twelve cranial nerves (CN) could be identified in all data sets (Figures 2A–E). However, in one individual of S23 measuring the diameter of the left trochlear and in one individual of S21 measuring the diameter of the right hypoglossal nerve was prevented due to extremely poor image contrast at the measuring position. In two further individuals (S23- and S23) the diameter of one of the hypoglossal nerves could not be measured, since it showed abnormal topology and did not pass the measuring position.

**FIGURE 3:**
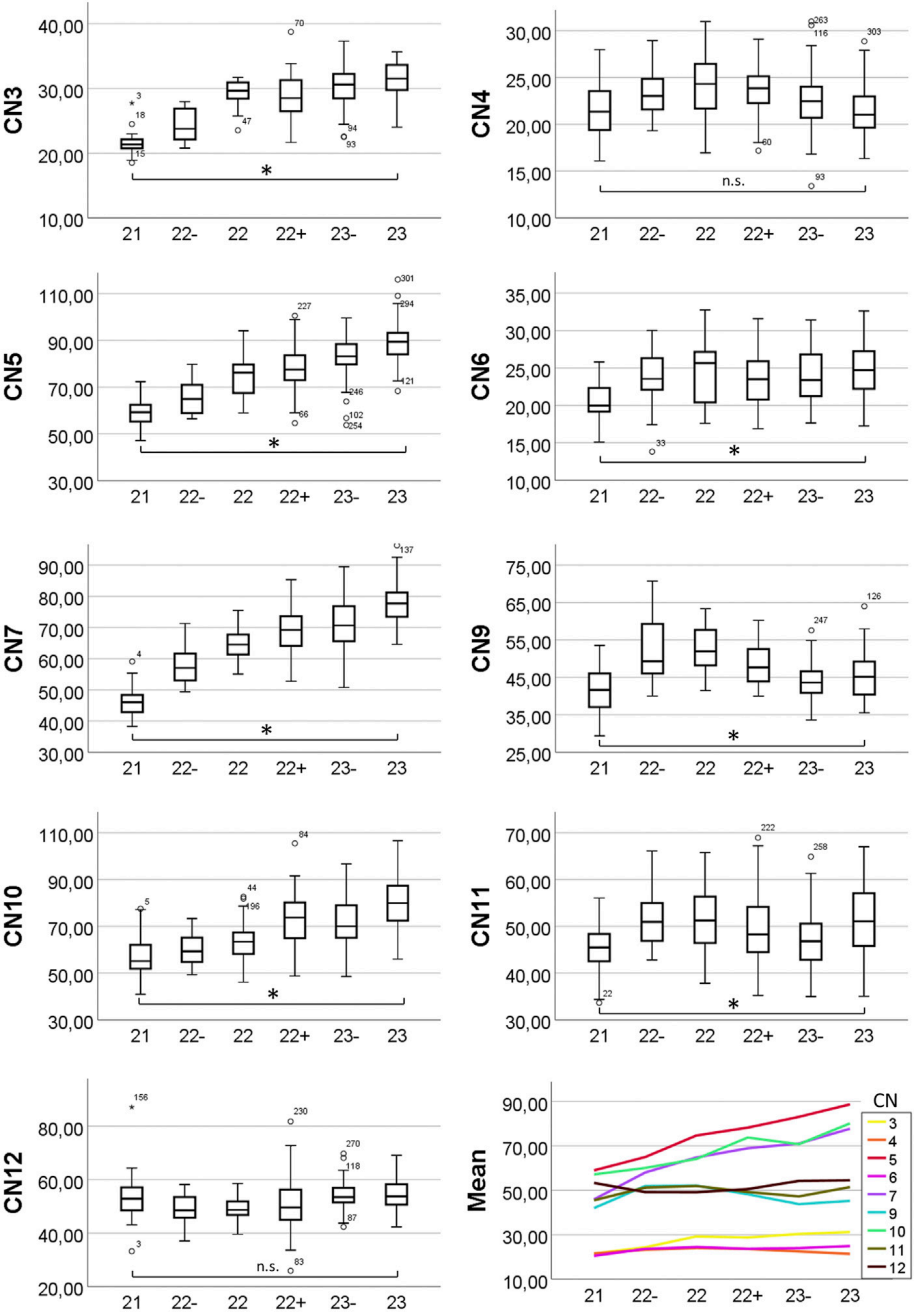
Box plots and means of measured nerve diameters. *Y*-axis showing diameter in micrometers, *x*-axis showing developmental stage. Changes in diameter were analyzed using one-way and Welch ANOVA followed by post-hoc analyses. *P* ≤ 0.05 was considered significant. Statistically significant diameter changes from S21 to S23 are indicated (asterisk). n. s. = not significant.

**FIGURE 4:**
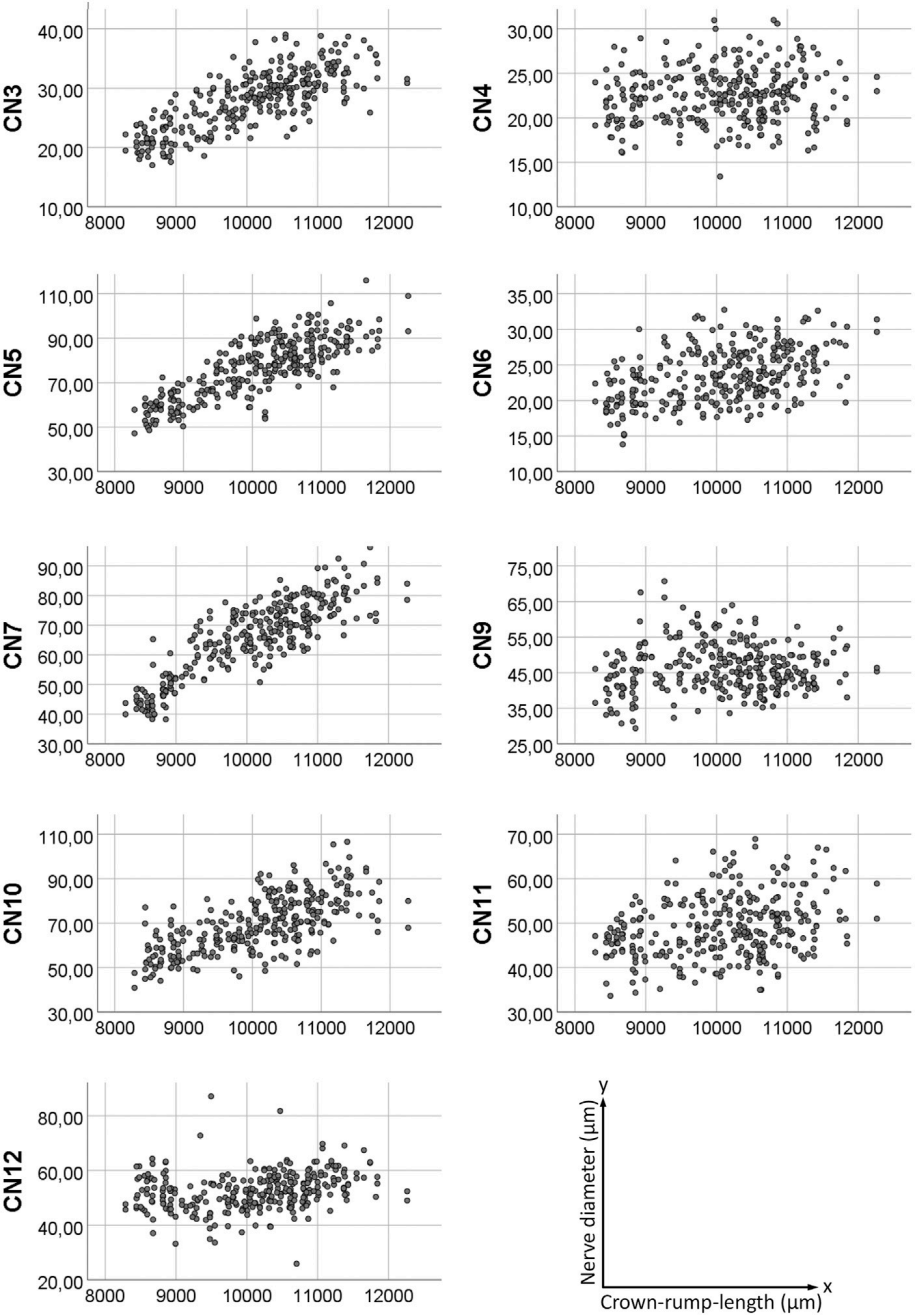
Scatter plot of measured nerve diameters. *Y*-axis showing nerve diameter in micrometers, *x*-axis showing crown-rump-length in micrometers.

Diameters measured for each developmental stage are provided in Table 1 and Figure 3. Measured diameters in respect of crown-rump-length (CRL) of the specimens are shown in Figure 4. The results of one-way ANOVA (CNs: 4, motor 5, 10), Welch ANOVA (CNs: 3, 6, 7, 9, 11, 12) and post-hoc analyses are displayed in Supplementary Table 1 and 2.

**TABLE 1:** Dimensions of cranial nerves at the developmental stages covered by E14.5. Data are based on a total of 152 individuals. Mean diameter and standard deviation (in parentheses) in µm. Positions of measurements are shown in Figure 4. *In four cases nerves could not be measured, which reduced the specimen number for these nerves: S21: CN12 (N = 53); S23-: CN12 (N = 63); S23: CN4 (N = 67), CN12 (N = 67).

	Position of measurement	Mean nerve diameter (std. deviation) in µm					
		S21 N = 54*	S 22-N = 24	S 22 N = 34	S 22 + N = 60	S 23-N = 64*	S 23 N = 68*
Oculomotor nerve (CN3)	at its exit from the parenchyma	21.5 (2.4)	24.3 (3.3)	29.2 (2.9)	28.7 (4.1)	30.3 (3.6)	31.3 (3.6)
Trochlear nerve (CN4)	within the forming dura mater	21.5 (2.8)	23.2 (2.6)	24.0 (3.3)	23.6 (2.6)	22.5 (3.3)	21.4 (2.7)
Motoric part of trigeminal nerve (CN5)	at its widest diameter within the trigeminal ganglion	58.8 (5.5)	65.0 (6.6)	74.7 (8.3)	78.2 (9.6)	83.0 (8.7)	88.6 (8.4)
Abducens nerve (CN6)	next to the internal carotid artery	20.4 (2.4)	23.6 (3.9)	24.4 (4.4)	23.7 (3.4)	24.0 (3.5)	24.8 (3.6)
Facial nerve (CN7)	next to the geniculate ganglion	45.9 (4.6)	58.0 (6.2)	64.8 (4.9)	68.9 (7.0)	71.1 (7.8)	77.6 (6.5)
Glossopharyngeal nerve (CN9)	caudal to the inferior ganglion	41.9 (6.2)	51.9 (8.7)	52.1 (5.8)	48.2 (5.2)	43.8 (5.0)	45.2 (5.7)
Vagus nerve (CN10)	caudal to the inferior ganglion	57.2 (8.3)	60.1 (6.7)	64.1 (8.4)	73.7 (10.2)	70.8 (10.0)	79.8 (10.6)
Accessory nerve (CN11)	at its widest diameter next to the superior ganglion of the vagus nerve	45.3 (4.9)	51.2 (6.0)	51.9 (7.1)	49.2 (7.1)	47.2 (5.8)	51.5 (7.4)
Hypoglossal nerve (CN12)	lateral to the forming hyoid	53.3 (7.7)	49.2 (5.3)	49.1 (4.4)	50.5 (9.3)	54.2 (5.4)	54.6 (5.5)

### Olfactory nerve (CN1)

In sagittal and axial sections, a plethora of small nerves, which emerged from the nasal septum and superior nasal turbinate could be traced to pass through the developing cribriform plate towards the olfactory bulb (Figures 2F,G). Inside the nasal cavity and also penetrating the cribriform plate small blood vessels ran between these nerves. In all individuals white matter covered the olfactory bulb. This continued as olfactory tract (Figure 2H).

### Optic nerve (CN2)

The material of the so called CN2 was part of the optic stalk. It was located inferior to the brain material of the optic recess, which extended from the floor of the third ventricle towards the optic cup (Figure 2I). CN2 formed the distinct innermost layer the optic cup and penetrated through its dorsal aspect. Caudal to the third ventricle they joined with the contralateral CN2 and formed a clearly distinguishable optic plate (Figure 2J), from where the optic tract could be followed towards the occipital part of the internal capsule (Figure 2K).

### Oculomotor nerve (CN3)

**FIGURE 5:**
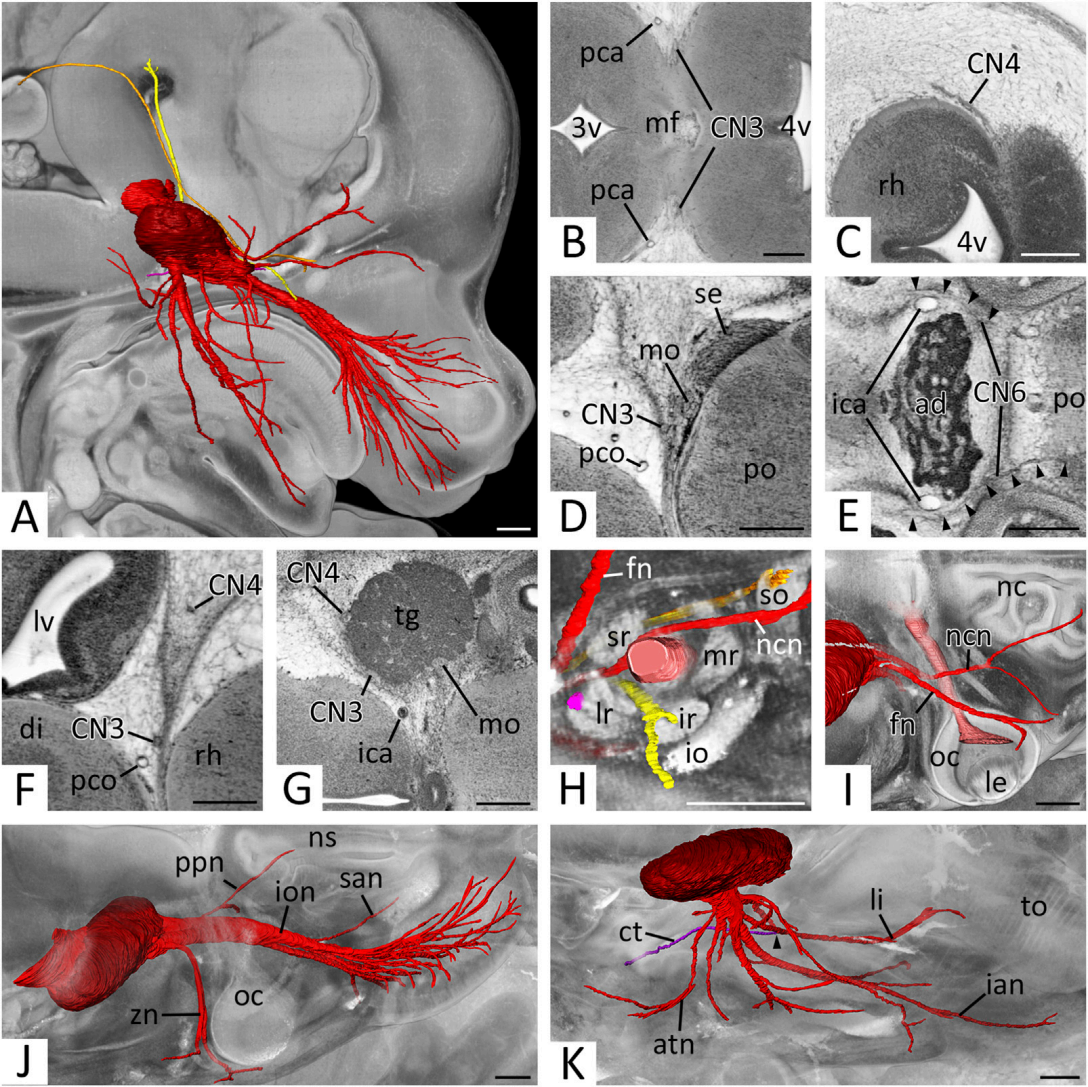
Cranial nerves (CN) 3 to 6: **(A)** Overview of right CNs 3, 4, 5 and 6.3D surface renderings in the context of a volume rendering of a mouse embryo of S23. View from right. CN3 (yellow), CN4 (orange), CN5 (red), trigeminal ganglion (dark red), CN6 (pink) **(B–E)** Exit of CN3 **(B)**, CN4 **(C)**, CN5 **(D)** and CN6 **(E)** in axial HREM sections. Anterior to the left, right on top. **(B)** Left and right CN3 emerging from the mesencephalic flexure (mf) as multiple rootlets that then start forming a solid nerve. **(C)** Right CN4 emerging from, and circumventing the rhombencephalon (rh). **(D)** Right motoric (mo) and sensory (se) root of CN5 emerging from the pons (po). Note the close proximity to CN3. **(E)** Both CN6s (arrowheads) at the level of the adenohypophysis (ad). Note the exit of the left CN6 from the caudal pons (po). Both CN6s run rostrally, lateral to the parasellar internal carotid artery (ica) **(F)** Caudal course of right CN3 and CN4 lateral to the brain. Axial HREM section. Anterior to the left, right on top **(G)** Right trigeminal ganglion (tg). Axial HREM sections. Anterior to the left, right on top. Note the adjacent CN3 and CN4 **(H)** Cranial nerves entering the right orbit. Surface renderings in the context of a volume rendering. View from lateral. CN2 (light red), CN3 (yellow), CN4 (orange), ophthalmic branches of CN5 (red), CN6 (pink). Note CN3 and CN4 directly inside their innervated muscles (lr, so) **(I–K)** Branches of the right trigeminal nerve. Surface renderings in the context of a volume rendering. View from cranio-lateral. **(I)** Ophthalmic branches (red) emerging from the trigeminal ganglion (dark red) and crossing CN2 (light red). Note the marks on the ganglion, where CN3 and CN4 are adjacent (compare panel G). **(J)** Maxillary branches (red) emerging from the trigeminal ganglion (dark red). **(K)** Mandibular branches (red) emerging from the trigeminal ganglion (dark red). Note the connection of the chorda tympani (ct) (violet) with the lingual nerve (li) (arrowhead). Abr. 3v = third ventricle, 4v = fourth ventricle, atn = auriculotemporal nerve, di = diencephalon, fn = frontal nerve, ian = inferior alveolar nerve, io = inferior oblique muscle, ion = infraorbital nerve, ir = inferior rectus muscle, le = lens, lr = lateral rectus muscle, lv = lateral ventricle, mr = medial rectus muscle, nc = nasal cavity, ncn = nasociliary nerve, ns = nasal septum, oc = optic cup, pca = posterior cerebral artery, pco = posterior communicating artery, ppn = pterygopalatine nerve, san = superior alveolar nerve, so = superior oblique muscle, sr = superior rectus muscle, to = tongue, zn = zygomatic nerve. Scale bars = 250 µm.

Many extremely thin rootlets left the brain at the concavity of the mesencephalic flexure and immediately fused to form CN3 posterior to the posterior cerebral artery (Figure 5B) Then it descended alongside this artery, the posterior communicating artery (Figure 5F), and finally the main stem, the internal carotid artery. With this it took its way towards the trigeminal ganglion, which it reached antero-medially at the level of the pituitary gland (Figure 5G). The nerve then entered the orbit (Figure 5H) and, in all data sets, could be traced towards the region underneath the forming eye. In optimally contrasted HREM data of all developmental stages nerve branches could be traced into all eye muscles known to be innervated by CN3.

The average diameter of CN3, at its exit from the cerebral parenchyma and thus immediately distal to the unification of the nerve rootlets that exit the brain (Figure 1A), in E14.5 was 28.1 (sd: 4.9) µm. Regarding to Geyer stages the minimal average diameter was 21.5 (sd: 2.4) µm in S21 embryos; the maximum average diameter was 31.3 (sd: 3.6) µm in S23 embryos. Statistics revealed a significant increase of the diameter from S21 to S23 (*p* < 0.001) (Figure 3).

### Trochlear nerve (CN4)

CN4 left the brain at the dorsolateral aspect of the mesencephalon lateral to the isthmus of the rhombencephalon (Figure 5C). It passed the mesencephalon laterally running in the mesenchyme destined to form the meninges and their cavities (Figure 5F) and reached the rostral aspect of the trigeminal ganglion (Figure 5G). At the level of the adenohypophysis it turned anteriorly and entered the orbit (Figure 5H) via the forming superior orbital fissure. In the orbit it crossed over the optic nerve and terminated in the superior oblique muscle. The last segment was particularly difficult to identify in all HREM data of all developmental stages.

The average diameter of the CN4, within the forming dura mater (Figure 1B) in E14.5 was 22.5 (sd: 3.0) µm. Regarding to Geyer stages the minimal average diameter was 21.5 (sd: 2.8) µm in S21 embryos and 21.4 (sd: 2.7) µm in S23 embryos; the maximum average diameter was 24.0 (sd: 3.3) µm in S22 embryos. Statistics showed no significant change of the diameter from S21 to S23 (*p* = 1.0) (Figure 3).

### Trigeminal nerve (CN5)

The motor and sensory roots of CN5 left respectively entered the brain lateral to the pons as two separate structures (Figure 5D). The tissue of the sensory root scattered almost immediately in the voluminous trigeminal ganglion, which was located lateral to the internal carotid artery. The motoric root passed as solid nerve through the ganglion material at its medio-posterior border (Figure 5G).

The average diameter of the motoric part of CN5 at its transition through the trigeminal ganglion (Figure 1C) in E14.5 was 76.7 (sd: 13.2) µm. Regarding Geyer stages the minimal average diameter was 58.8 (sd: 5.5) µm in S21 embryos; the maximal average diameter was 88.6 (sd: 8.4) µm in S23 embryos. Statistics revealed a significant increase of the diameter from S21 to S23 (*p* < 0.001) (Figure 3).

The ganglion formed clearly distinguishable maxillary and mandibular nerves as well as two solitary small nerves travelling into the orbit. The sum of the latter was considered as ‘ophthalmic nerve’.

### “Ophthalmic nerve’ (CN5_1_)

A single CN5_1_ did not exist. Instead, a separated frontal and nasociliary nerve emerged directly from the ganglion material (Figure 5I). Both entered the orbit (Figure 5H), the frontal nerve near CN4, the nasociliary nerve near CN3. A branch towards the forming lacrimal gland could not be identified with certainty, however the zygomaticofacial branch of the zygomatic nerve (CN5_2_) passed the gland in close proximity. Inside the orbit the frontal and nasociliary nerves ran towards the medial aspect of the orbit, with the frontal nerve crossing over the superior eye muscles and the nasociliary nerve crossing over CN2. A branch of the latter, which entered the nasal cavity could be identified in all data sets. Further branches to the ciliary ganglion could not be distinguished with certainty in the HREM data.

### Maxillary nerve (CN5_2_)

CN5_2_ emerged from the frontal aspect of the trigeminal ganglion and formed four branches, which we identified as zygomatic, pterygopalatine, superior alveolar and infraorbital nerves (Figure 5J). The zygomatic nerve was always traceable towards the skin latero-caudal to the eye closely passing by the forming lacrimal gland. The pterygopalatine nerve branched quickly. Its branches could be traced towards the nasal septum and palate. The superior alveolar nerve could be traced to the primordium of the upper incisor tooth and a rather thick infraorbital nerve ran towards the lateral nasal capsule, formed an ansa around the infraorbital artery and split into numerous branches, which terminated in the skin and the follicles of the vibrissae. In optimally contrasted image data communicating branches between the forming pterygopalatine ganglion and the branches of the maxillary nerve could be observed.

### Mandibular nerve (CN5_3_)

CN5_3_ formed at the inferior aspect of the trigeminal ganglion. It received some material from the trigeminal ganglion and all of the motor portion. The nerve soon formed several short nerve bundles that could be traced towards the medial pterygoid, mylohyoid and masseter muscles. It then gave rise to long bundles, which we identified as auriculotemporal, lingual and inferior alveolar nerves (Figure 5K). The auriculotemporal nerve passed Meckel’s cartilage frontally and reached the skin anterior to the external acoustic meatus. The lingual nerve connected with a small nerve bundle we identified as chorda tympani. It then travelled medially to Meckel’s cartilage towards the tongue and entered it from its base. The inferior alveolar nerve travelled laterally to Meckel’s cartilage. In individuals of S22- and older, in which ossification had already started, the later intracanalicular segment of the nerve was surrounded by the trabecular tissue of the forming bone. From the proximal segment the mylohyoid branch split off. It could be traced towards the anterior belly of the digastric muscle. The nerve finally terminated in the skin of the lower jaw and at the primordium of the lower incisors.

### Abducent nerve (CN6)

CN6 left the brain at the caudal pons and straightly ran towards the pituitary gland, which it passed laterally to the parasellar segment of the internal carotid artery (Figure 5E). It then entered the orbit (Figure 5H), where it crossed under CN3 and the nasociliary nerve to enter the lateral rectus muscle.

The average diameter of CN6 next to the internal carotid artery (Figure 1D) in E14.5 was 23.5 (sd: 3.7) µm. Regarding Geyer stages the minimal average diameter was 20.4 (sd: 2.4) µm in S21 embryos; the maximal average diameter was 24.8 (sd: 3.6) µm in S23 embryos. Statistics revealed a slight but significant increase of the diameter from S21 to S23 (*p* < 0.001) (Figure 3).

### Facial nerve (CN7)

**FIGURE 6:**
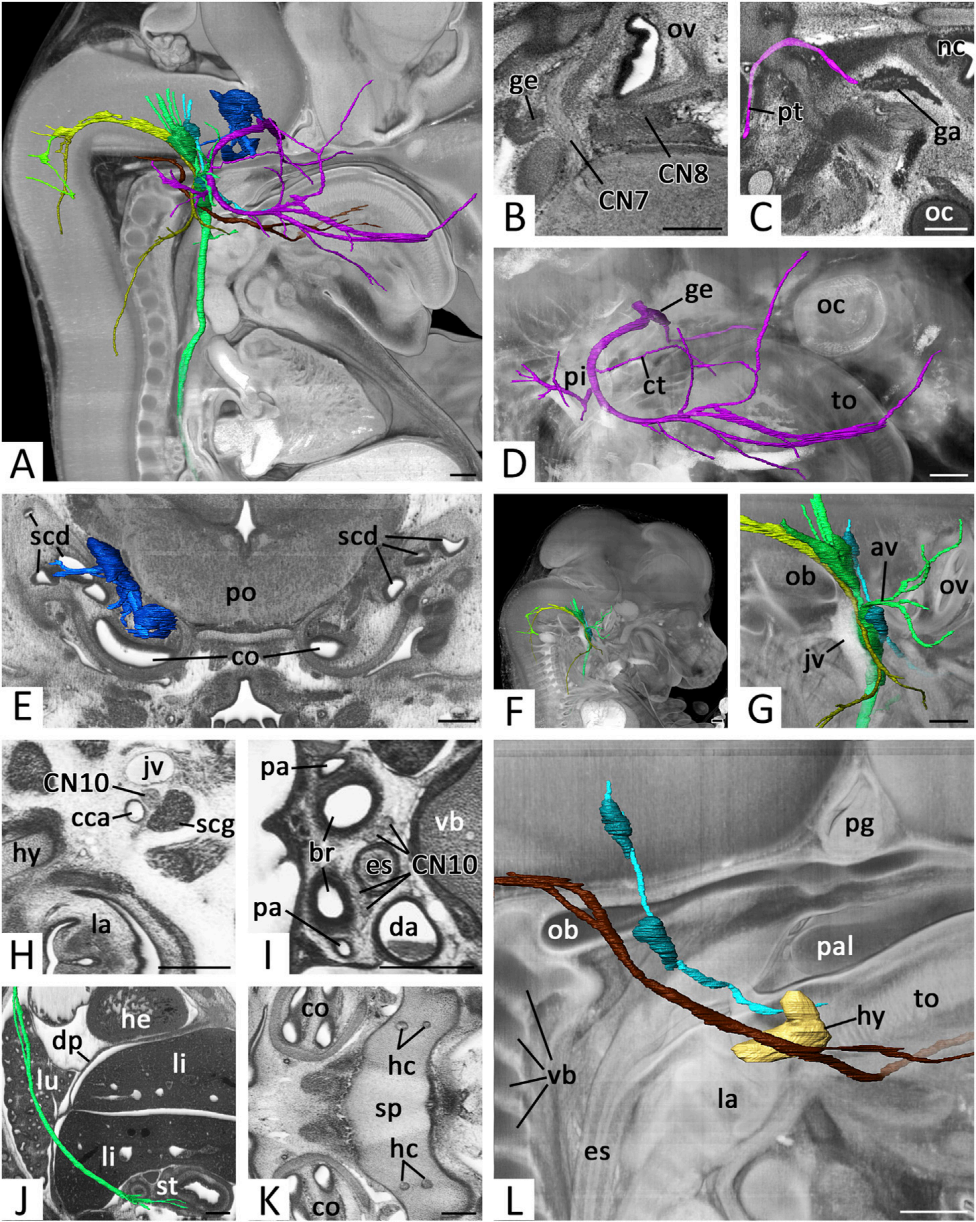
Cranial nerves (CN) 7 to 12: **(A)** Overview of right CNs 7, 8, 9, 10, 11 and 12.3D surface renderings in the context of a volume rendering of a mouse embryo of S23. View from right. CN6 (pink), CN7 (violet), geniculate ganglion (dark violet), CN8 (blue), ganglia CN8 (dark blue), CN9 (turquoise), ganglia of CN9 (dark turquoise), CN10 (green), ganglia of CN10 (dark green) CN11 (olive), cranial root CN11 (light green), CN12 (brown) **(B)** Exit of right CN7 and CN8 in an axio-frontal HREM resection. Anterior to the left, right on top **(C)** Right greater petrosal nerve (pt) (pink) reaching the ganglion mass in the pterygopalatine fossa (ga). Surface renderings in the context of an axial HREM section. Anterior to the right, left on top **(D)** Right facial nerve (CN7) (violet) with geniculate ganglion (ge) (dark violet). Surface renderings in the context of a volume rendering. View from right **(E)** Right vestibulocochlear nerve (CN8) (blue) and its ganglia (dark blue). Surface renderings in the context of frontal HREM resection **(F,G)** Right CNs 9 (turquoise), 10 (green) and 11 (olive) passing through the jugular foramen. Surface renderings in the context of a volume rendering. View from right **(G)** Shows magnification from **(F)**. Note the superior and inferior ganglia (dark turquoise, dark green) and the topology of the jugular vein (jv) **(H–J)** Course of the right vagus nerve (CN10) (green). Axial HREM sections, anterior to the left and right on top **(H,I)** and surface rendering in the context of a sagittal resection, anterior to the right. **(H)** Cervical CN10 between common carotid artery (cca), jugular vein and superior cervical sympathetic ganglion (scg). **(I)** Thoracic CN10s lateral to the esophagus (es) **(J)** Thoracic and abdominal CN10 (green) reaching the stomach (st) **(K)** Hypoglossal canals (hc) in the sphenoid bone (sp). Axial HREM section. Anterior to the left, right on top **(L)** Right CNs 9 (turquoise) and 12 (brown) in the context of the forming hyoid bone (hy) (light brown) reaching the tongue (to). Surface renderings in the context of a volume rendering. View from right. Abr. av = auricular branches of vagus nerve, br = bronchus, co = cochlea, ct = chorda tympani, da = descending aorta, dp = diaphragm, he = heart, la = larynx, li = liver, lu = lung, nc = nasal cavity, ob = occipital bone, oc = optic cup, ov = otic vesicle, pa = pulmonary artery, pal = palate, pg = pituitary gland, pi = pinna, po = pons, scd = semicircular duct, vb = vertebral body. Scale bars = 250 µm.

The facial nerve (CN7) left the lateral rhombencephalon and, in a semicircle, circumvented the cochlea frontally. Near the outer aspect of the apogee of this curve and immediately posterior to the trigeminal ganglion the geniculate ganglion could be discerned in all specimens (Figure 6B).

Proximal to the geniculate ganglion the greater petrosal nerve emerged from CN7 as a thin branch. It travelled medio-caudally to the lateral aspect of the internal carotid artery. Here it received additional nerve material and turned rostrally to reach a voluminous mass of distributed ganglion cells in the pterygopalatine fossa (Figure 6C).

After having passed the geniculate ganglion, the stem of CN7 turned posteriorly. It crossed under the stapedial artery and passed Reichert’s cartilage, where a branch, we identified as the chorda tympani, split off and ran towards the lingual nerve. Other branches could be always traced towards the auricle and the posterior belly of the digastric muscle. The stem continued towards the fronto-lateral side of the head where it ramified into several branches. Some of them could be followed to enter the facial muscles. (Figure 6D).

The average diameter of CN7 next to the geniculate ganglion (Figure 1E) in E14.5 was 65.9 (sd: 12.4) µm. Regarding Geyer stages the minimal average diameter was 45.9 (sd: 4.6) µm in S21 embryos; the maximal average diameter was 77.6 (sd: 6.5) µm in S23 embryos. Statistics revealed a significant increase of the diameter from S21 to S23 (*p* < 0.001) (Figure 3).

### Vestibulocochlear nerve (CN8)

As CN8 we identified nerve branches connecting the lateral rhombencephalon and an irregularly shaped mass of ganglion cells in the center of the otic vesicle (Figure 6B). Branches from the ganglion to the lateral semicircular canals, utricle and saccules could be identified in embryos of all stages, but only if HREM data had optimal contrasts (Figure 6E).

### Glossopharyngeal nerve (CN9)

CN9 emerged with several roots from the lateral rhombencephalon. It descended towards the forming jugular foramen (Figures 6F,G). Near the foramen, two ganglia were tagged to the nerve. One intra- (superior ganglion) and one extra cranially (inferior ganglion). The material was in close proximity to the ganglions tagged to the vagus nerve. Independent from the developmental stage, allocation of the ganglion material to CN9 and CN10 was challenging. Furthermore, the inferior ganglion mass was in very close proximity with the material of the sympathetic superior cervical ganglion. However, a clear distinction of the superior cervical ganglion was always possible by carefully analyzing the full resolution HREM data in a careful image by image mode.

Distal to the inferior ganglion, CN9 passed the internal carotid artery anteriorly, crossed under the stylopharyngeus muscle and ran medially to the primordial cartilage of the hyoid bone to the root of the tongue (Fig. 5L). Only the main stem of CN9 could be traced. Nerves, splitting from CN9 to reach their targets in the head and neck could not be followed with a sufficient amount of certainty.

The average diameter of CN9 caudal to its inferior ganglion (Figure 1F) in E14.5 was 46.2 (sd: 6.8) µm. Regarding Geyer stages the minimal average diameter was 41.9 (sd: 6.2) µm in S21 embryos; the maximal average diameter was 52.1 (sd: 5.8) µm in S22 embryos. Statistics revealed a slight but significant increase of the diameter from S21 to S23 (*p* = 0.033) (Figure 3).

### Vagus nerve (CN10)

CN10 left the lateral hindbrain immediately occipital to CN9. It ran towards the forming jugular foramen (Figures 6F,G) and entered the ganglion masses located superior and inferior to it (compare to CN9).

Distal to the superior ganglion, a nerve split from CN10 and turned laterally. We identified it as auricular branch. It crossed the facial nerve dorsally, split and terminated in the area of the forming external acoustic meatus and auricle. Distal to the inferior ganglion multiple branches left CN10 and travelled towards the pharynx.

The main stem of CN10 continued in the neck, running between the internal jugular vein and the common carotid artery (Figure 6H). At the level of the cervicothoracic junction the nerve crossed the subclavian artery ventrally. The right nerve then immediately turned dorsally to reach the right side of the esophagus at the level of the tracheal bifurcation. The left CN10 continued to reach the junction of ductus arteriosus and descending aorta. Here it also turned dorsally and joined the left side of the esophagus at the level of the tracheal bifurcation.

The nerves then continued to run caudally left and right to the esophagus (Figure 6I). In many embryos of S22 and older the left and right sided CN10 shifted ventrally, to run for a short distance side by side ventral to the esophagus. In the caudal thoracic cavity, the left CN10 then gradually shifted ventrally and the right CN10 dorsally to the esophagus. In this relationship, they passed the diaphragm, before they split into numerous branches covering the anterior and posterior face of the stomach (Figure 6J).

When turning around the right subclavian artery and the ductus arteriosus respectively, the CN10s gave rise to the recurrent laryngeal nerve. This immediately turned cranially and ran laterally to the trachea. It could be traced passing the thyroid gland dorsally and then vanish when entering the pharyngeal muscles.

The average diameter of CN10 caudal to its inferior ganglion (Figure 1G) in E14.5 was 69.4 (sd: 12.4) µm. Regarding Geyer stages the minimal average diameter was 57.2 (sd: 8.3) µm in S21 embryos; the maximal average diameter was 79.8 (sd: 10.6) µm in S23 embryos. Statistics revealed a significant increase from S21 to S23 (*p* < 0.001) (Figure 3).

### Accessory nerve (CN11)

CN11 is formed by unification of a spinal and cranial root. Both roots could be identified in all data sets. The spinal root emerged from the cranial segments of the cervical spinal cord. They ascended laterally by forming a single strand, which passed through the foramen magnum. In individuals of S22 and older ganglion material lay close to the spinal root.

Inside the skull the cranial root left the rhombencephalon. They unified and headed for the jugular foramen. Before reaching it the cranial root met the spinal root. Once at the foramen, the nerve material of CN11 mingled with that of CN9 and CN10 (Figures 6F,G).

At the level of the inferior ganglia of CN9 and CN10, branches of CN11 turned laterally. They passed the jugular vein ventrally and entered the sternocleidomastoid muscle. After penetrating this muscle, they passed the jugular lymphatic sac ventrally and reached the trapezius muscle. Along its ventral side the remaining nerve then ran caudally and finally vanished between the muscle fibers.

The average diameter of CN11 (respectively its external ramus) next to the superior ganglion of the vagus nerve (Figure 1H) in E14.5 was 49.1 (sd: 6.9) µm. Regarding Geyer stages the minimal average diameter was 45.3 (sd: 4.9) µm in S21 embryos; the maximal average diameter was 51.9 (sd: 7.1) µm in S22 embryos. Statistics revealed a slight but significant increase of the diameter from S21 to S23 (*p* < 0.001) (Figure 3).

### Hypoglossal nerve CN12

CN12 was formed by multiple nerve roots that left the occipital segments of the rhombencephalon. They joined and passed through the occipital bone in one or two, in rare cases three, hypoglossal canals (Figure 6K). Outside the skull all roots fused to form a single nerve. Taking a horizontal course rostrally, it crossed the jugular vein. In 84% it bilaterally passed the vein medially. In 6% it bilaterally passed laterally. In 10% one of the nerves crossed the vein medially, while the contralateral passed laterally.

CN12 then crossed the carotid artery at the level of the bifurcation and slightly ascended to pass the hyoid bone immediately lateral to its greater horn. Finally, it was joined by the lingual artery. Together they entered the tongue running between the styloglossus and hyoglossus muscles (Fig. 6L).

The average diameter of CN12 lateral to the forming hyoid bone (Figure 1I) in E14.5 was 52.4 (sd: 7.0) µm. Regarding Geyer stages the minimal average diameter was 49.1 (sd: 4.4) µm in S22 embryos; the maximal average diameter was 54.6 (sd: 5.5) µm in S23 embryos. Statistics showed no significant change of the diameter between S21 and S23 (*p* = 0.893) (Figure 3).

### Cranial nerve abnormalities in mutants

In order to demonstrate the relevance of the presented descriptive and metric data, we analyzed the cranial nerves of homozygous offspring of 4 DMDD KO-lines.

**FIGURE 7:**
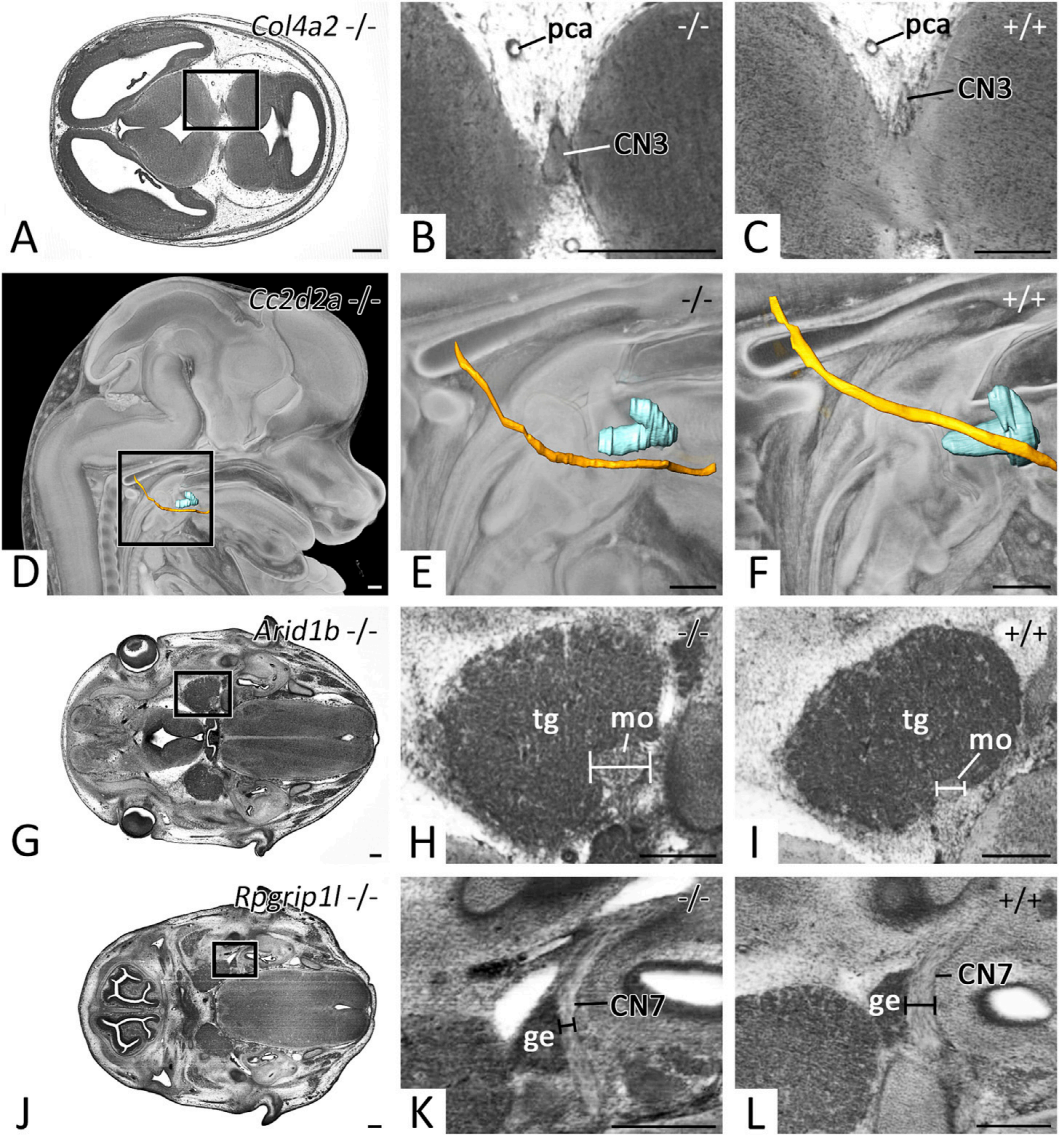
Cranial nerve abnormalities in homozygous offspring of embryonically or perinatally lethal KO-lines. Axial HREM sections. Anterior to the left, right on top **(A–C, G–L)**. Surface renderings in the context of a volume rendering. View from right **(D–F) (A–C)** Abnormal thickening of CN3 in a *Col4a2 −/−* mutant **(A,B)**, **(C)** serves as control **(D–F)** Abnormal topology of CN12 (yellow) in respect to the hyoid bone (turquoise) in a *Cc2d2a −/−* mutant **(D,E)**, **(F)** serves as control **(G–I)** Abnormal thickening of the motoric part (mo) of CN5 in an *Arid1b* −/− mutant **(G,H)**, **(I)** serves as control **(J-L)** Abnormal thinning of CN7 in an *Rpgrip1l −/−* mutant **(J,K,L)** serves as control. Abr. ge = geniculate ganglion, pca = posterior cerebral artery, tg = trigeminal ganglion. Scale bars = 250 µm.

Besides other malformations, mutants of the *Col4a2* and *Cc2d2a* KO-lines featured abnormal cranial nerve topology. In 4 of 4 *Col4a2* mutants two thick roots instead of multiple thin rootlets left the brain to form CN3 (Figures 7A,B). After fusing, the main stem coursed and branched regularly. 3 of 4 *Cc2d2a* mutants showed abnormal topology of CN12. It passed the hyoid bone caudally, instead of laterally (Figures 7D,E).

In mutants of the *Arid1b* and *Rpgrip1l* KO-lines cranial nerves of abnormal thickness were detected. 3 of 7 *Arid1b* mutants featured thickening of the motoric portion of CN5 (Figures 7G,H), while 1 of 3 *Rpgrip1l* mutants showed thinning of CN7 (Figures 7J,K).

## Discussion

Phenotyping genetically engineered mouse embryos/fetuses is an essential step in researching the function of genes; for studying the mechanisms, which steer regular development, growth and tissue remodeling; and for examining the role genes play in the genesis of pathologies. Comprehensive reference data are the key for correct diagnosis of phenotype abnormalities. Ideally they are based on a significant number of individuals, describe the regular anatomy in high detail, define the spectrum of norm variants, and present objective metric characterizations of organs and important anatomic structures.

Existing reference data describing the phenotype of mouse embryos are often based on 2D sections (Kaufman, 1992), wherefore they poorly suit for providing topological information and fail to provide spatial details on tissue level. Furthermore, they are often based on small numbers of individuals and therefore do not include sound metric data or information on anatomic norm variations. This is especially problematic for diagnosing defects of the spatially highly complex cardiovascular and nervous systems and for using topological features and measurements of vessel diameters and nerve thicknesses as diagnostic parameters. To compensate the lack of meaningful 3D reference data, we recently started producing highly detailed descriptions of the organ systems of large numbers of mice of E14.5. E14.5 was selected, because it is the time in which organogenesis is largely finished and fetal life begins.

In a first effort, we produced reference data for diagnosing abnormalities of the cardiovascular system (Geyer et al., 2017b, 2022). The recent study continues these efforts and focused on providing high detailed anatomic descriptions and metric data of cranial nerves. It is based on careful analysis of high resolution digital volume data of around 150 individuals and acknowledges the different morphology embryos harvested at E14.5 exhibit.

To demonstrate the capacity of the descriptions to serve as objective references for screening the cranial nerve phenotype of genetically compromised and engineered mutants, we immediately employed the reference data for screening mutants produced in the DMDD project. In particular, we used them as basis for identifying metric and topologic abnormalities of CN3, CN5, CN7 and CN12 in mutants of the *Col4a2*, *Cc2d2a*, *Arid1b* and *Rpgrip1l* lines. Although the detected features do not appear to be spectacular at a first glance, they might have serious consequences. The diagnosed neuroma of the motor portion of CN5 and the thinning of CN7 could hinder feeding due to insufficient function of masticatory and mimic muscles - if still present in the perinatal period. This might in turn cause perinatal death or consumption of the pups by the mother. Similar patterns were also observed in studies investigating cranial nerve differentiation in *LgDel* mutant lines, a mouse model for DiGeorge (22q11.2) deletion syndrome (Karpinski et al., 2014; Maynard et al., 2020). Also, the abnormal topology of CN12 seen in the *Cc2d2a* −/− mutant is of high importance. Although *per se* not a pathology, this feature was recently identified as a highly penetrant marker for life threatening central nervous system defects of low penetrance in littermates of the same KO-line (Reissig et al., 2021b).

Several concepts for classifying the cranial nerves were proposed (Davis et al., 2014; Martinez-Marcos and Sanudo, 2019). Some emphasize developmental, some functional aspects. The traditional classification is simply descriptive and distinguishes twelve pairs numbered according to the sequence of their exit from brain tissue and their transition through the meninges as cranial nerve 1 to 12. To make our data as widely comparable as possible, we stringently used this traditional anatomical classification, which is also used in the Terminologia Neuroanatomica edited by the International Federation of Associations of Anatomists (FIPAT, 2017). Hence, in this study, we consider the olfactory nerve as the sum of the fila olfactoria and the optic nerve as the segment of the diencephalic retinal fibers in between the retina, which in embryos forms in the optic cup and the optic plate, which is the embryonic pendant to the optic chiasm. We further accept the facial and intermediate (Wrisberg’s) nerve as a single CN7, the accessory nerve as being formed by two radices and branching into two rami, and CN11 and 12 as true cranial nerves.

Our study provides systematic, 3D characterizations of cranial nerve trajectories in the context of the overall fetal anatomy. It shows, that in principle, the gross anatomy and branching patterns of the nerves of mouse embryos and adult humans are comparable. Exceptions are obvious and predictable: Naturally, the nerves of embryos are much smaller, since spreading of new nerve fibers along the established routes continues till the end of the neonatal period (Kaplan et al., 2009). Also, embryos still feature optic stalks with optic recesses and optic cups. In addition, the brain features the embryonic flexures, which effects the exit pattern of the cranial nerves. E14.5 mice have an acute mesencephalic flexure and we consider this as a reason, for CN3 exiting the brain not as a single strand, but as several rootlets, which join later in the meningeal tissue. Like the brain, the meninges and meningeal spaces as well as the skull base with its foramina and associated structures have not yet reached their final shape. Hence the cranial nerves do not yet pass a subarachnoid space or have reached their final relationships inside the intracranial extradural spaces.

Three cranial nerves, CN1, CN5 and CN11 show features, which we consider as related to fundamental anatomic differences between the two species, mouse and man. CN1: As the olfactory system in mice is much more pronounced than in humans, the number of olfactory fibers innervating the olfactory mucosa is very high; CN5: The mouse receives important sensory input from a large number of vibrissae. They are innervated by branches of the maxillary nerve (Figure 4J), which do not exist in man; CN11: In E14.5 mice, ganglion material is located along the spinal root of the accessory nerve. This material does not exist in humans and is entirely unexpected, since CN11 is considered as a motor nerve. Further studies are required to characterize this material and to examine its fate.

Mouse embryos harvested at E14.5 differ significantly in respect to crown rump length and other markers of maturity. Thus, comparing the morphology without acknowledging the maturity might result in false interpretation of phenotype features. To cope with this, a staging system was introduced by Geyer et al., which distinguishes six stages (S21-S23) of maturation in E14.5 mice (Geyer et al., 2017c). Relying on this system is of very high importance, since gene knockouts often cause impaired metabolic situations, which result in developmental delay. Hence, they quite frequently are staged as S21 or S22-. To diagnose phenotype abnormalities, they must be compared with wild types also staged as S21 or S22. As our data show, some cranial nerves increase their diameter (CN5, CN7) from S21 to S23, some keep it roughly constant (CN4, CN6, CN9, CN11, CN12) and some increase it in early stages, while keeping it roughly constant during later stages (CN3, CN10). Thus, it is vital to consider the precise developmental stage when attempting to diagnose neuromas or abnormal thin nerves.

The basis of our reference data are digital volume data with isotropic voxel dimensions of 3 µm produced with the High Resolution Episcopic Microscopy (HREM) method (Weninger et al., 2006, 2014). In our opinion the use of HREM is ideal for being able to diagnose cranial nerve abnormalities in E14.5 mice (Adams et al., 2013). In contrast to techniques such as µCT or µMRI commercial HREM machines are relatively cost efficient in acquisition. Preparing the specimens for data generation is based on standard laboratory methods, specialized skills for embedding and operating the machine can be obtained within a few days by experienced lab technicians. Yet, expert anatomical knowledge is required for profound analysis of the data. Basic HREM setup and specimen preparation are described in various publications (Geyer et al., 2017a; Weninger and Geyer 2021). HREM is a histologic technique, which produces stacks of inherently aligned digital images, which are of almost the quality of images of histological sections. But, in contrast to conventional histological section images, HREM images are captured during physically sectioning of resin embedded specimens on a microtome. Therefore, they are perfectly aligned and can be quickly and easily stacked to be converted to a digital volume data set. That being said, the data provides morphological information. Therefore, nerve and ganglion material could be distinguished by their texture. The correctness of the interpretation was backed by comparisons with histological images (Kaufman, 1992). In addition, HREM data have limitations common to all histological imaging approaches (Reissig et al., 2021a). The most significant is that the specimens have to be dehydrated, infiltrated and embedded in resin. This causes shrinkage of about 15%, which has to be taken into consideration when using measurements as reference for diagnosing pathologies in mutants imaged with alternative imaging techniques. Also staining artefacts may occur, which obscure information. HREM is capable of visualizing the exact topology of all cranial nerves in context of overall morphology of whole mouse fetuses. In embryos of earlier stages, sophisticated confocal approaches have been used to trace individual sensory and motor axons of cranial nerves after specific labeling. These studies have provided further insight into basic mechanisms underlying abnormal growth and sprouting of the cranial nerves (Motahari et al., 2020). However, these techniques are limited to early, transparent mouse embryos. Yet, as discussed in several publications (Norris et al., 2013; Weninger et al., 2014; Wilson et al., 2016) HREM provides very good results in phenotyping E14.5 embryos and its data quality is superior to many alternatives.

## Data Availability

The raw data supporting the conclusion of this article will be made available by the authors, without undue reservation.
