# Supplementary material for: Detailed characterizations of cranial nerve anatomy in E14.5 mouse embryos/fetuses and their use as reference for diagnosing subtle, but potentially lethal malformations in mutants
Source: Front Cell Dev Biol. 2022 Nov 9;10:1006620. doi: 10.3389/fcell.2022.1006620 (PMC9682249; doi:10.3389/fcell.2022.1006620)
Supplement: Supplementary file 3 [file DataSheet1.PDF]

Supplementary Material

Supplementary Table 1. Results of one-way ANOVA (CNs 4, 5 and 10) and Welch ANOVA (CNs 3, 6, 7, 9, 11, and 12), depending on the homogeneity of variances, to assess stage dependent effects on nerve diameter. p ≤ 0,05 is considered statistically significant.

	**One-way ANOVA**			
CN	df (between groups)	df (within groups)	F	Sig.
CN4	5	297	7,245473	< 0,001
CN5	5	298	99,813155	< 0,001
CN10	5	298	43,402105	< 0,001
	**Welch ANOVA**			
CN	df1	df2	Statistic	Sig.
CN3	5	91,828	113,826	< 0,001
CN6	5	18,261	110,357	< 0,001
CN7	5	241,436	114,422	< 0,001
CN9	5	19,300	109,703	< 0,001
CN11	5	9,883	112,698	< 0,001
CN12	5	9,563	114,838	< 0,001

Supplementary Table 2. Results of Tukey and Games-Howell post-hoc analyses between developmental stages of E14.5: S21, S22-, S22, S22+ and S23. p ≤ 0,05 is considered statistically significant. * Mean difference is significant.

	**Post-Hoc Analysis (Tukey)**				
CN	(I) Stage	(J) Stage	Mean Difference (I-J)	Std. Error	Sig.
**CN4**	S21	S22-	-1,74940	,70410	,132
		S22	-2,53570*	,62834	,001
		S22+	-2,10015*	,53835	,002
		S23-	-,98185	,53032	,434
		S23	,08138	,52486	1,000
	S22-	S21	1,74940	,70410	,132
		S22	-,78630	,76517	,908
		S22+	-,35075	,69318	,996
		S23-	,76755	,68696	,874
		S23	1,83078	,68275	,082
	S22	S21	2,53570*	,62834	,001
		S22-	,78630	,76517	,908
		S22+	,43555	,61608	,981
		S23-	1,55385	,60907	,113
		S23	2,61708*	,60433	< 0,001
	S22+	S21	2,10015*	,53835	,002
		S22-	,35075	,69318	,996
		S22	-,43555	,61608	,981
		S23-	1,11830	,51574	,256
		S23	2,18153*	,51012	< 0,001

	S23-	S21	,98185	,53032	,434
		S22-	-,76755	,68696	,874
		S22	-1,55385	,60907	,113
		S22+	-1,11830	,51574	,256
		S23	1,06323	,50164	,280
	S23	S21	-,08138	,52486	1,000
		S22-	-1,83078	,68275	,082
		S22	-2,61708*	,60433	< 0,001
		S22+	-2,18153*	,51012	< 0,001
		S23-	-1,06323	,50164	,280
**CN5**	S21	S22-	-6,16037*	1,99646	,027
		S22	-15,87125*	1,78164	< 0,001
		S22+	-19,40370*	1,52649	< 0,001
		S23-	-24,20021*	1,50373	< 0,001
		S23	-29,79802*	1,48335	< 0,001
	S22-	S21	6,16037*	1,99646	,027
		S22	-9,71088*	2,16962	< 0,001
		S22+	-13,24333*	1,96550	< 0,001
		S23-	-18,03984*	1,94787	< 0,001
		S23	-23,63765*	1,93219	< 0,001
	S22	S21	15,87125*	1,78164	< 0,001
		S22-	9,71088*	2,16962	< 0,001
		S22+	-3,53245	1,74688	,332
		S23-	-8,32896*	1,72703	< 0,001
		S23	-13,92676*	1,70931	< 0,001

	S22+	S21	19,40370*	1,52649	< 0,001
		S22-	13,24333*	1,96550	< 0,001
		S22	3,53245	1,74688	,332
		S23-	-4,79651*	1,46238	,015
		S23	-10,39431*	1,44142	< 0,001
	S23-	S21	24,20021*	1,50373	< 0,001
		S22-	18,03984*	1,94787	< 0,001
		S22	8,32896*	1,72703	< 0,001
		S22+	4,79651*	1,46238	,015
		S23	-5,59780*	1,41729	,001
	S23	S21	29,79802*	1,48335	< 0,001
		S22-	23,63765*	1,93219	< 0,001
		S22	13,92676*	1,70931	< 0,001
		S22+	10,39431*	1,44142	< 0,001
		S23-	5,59780*	1,41729	,001
**CN10**	S21	S22-	-2,89005	2,34088	,820
		S22	-6,95875*	2,08900	,012
		S22+	-16,58263*	1,78984	< 0,001
		S23-	-13,60385*	1,76315	< 0,001
		S23	-22,65169*	1,73925	< 0,001
	S22-	S21	2,89005	2,34088	,820
		S22	-4,06870	2,54392	,600
		S22+	-13,69258*	2,30458	< 0,001
		S23-	-10,71380*	2,28392	< 0,001

		S23	-19,76164*	2,26552	< 0,001
	S22	S21	6,95875*	2,08900	,012
		S22-	4,06870	2,54392	,600
		S22+	-9,62388*	2,04825	< 0,001
		S23-	-6,64510*	2,02497	,015
		S23	-15,69294*	2,00420	< 0,001
	S22+	S21	16,58263*	1,78984	< 0,001
		S22-	13,69258*	2,30458	< 0,001
		S22	9,62388*	2,04825	< 0,001
		S23-	2,97878	1,71467	,508
		S23	-6,06906*	1,69009	,005
	S23-	S21	13,60385*	1,76315	< 0,001
		S22-	10,71380*	2,28392	< 0,001
		S22	6,64510*	2,02497	,015
		S22+	-2,97878	1,71467	,508
		S23	-9,04784*	1,66179	< 0,001
	S23	S21	22,65169*	1,73925	< 0,001
		S22-	19,76164*	2,26552	< 0,001
		S22	15,69294*	2,00420	< 0,001
		S22+	6,06906*	1,69009	,005
		S23-	9,04784*	1,66179	< 0,001
	**Post-Hoc Analysis (Games-Howell)**				
**CN3**	S21	S22-	-2,78796*	,75249	,009
		S22	-7,67502*	,59266	< 0,001
		S22+	-7,20113*	,62947	< 0,001

		S23-	-8,72874*	,56076	< 0,001
		S23	-9,79635*	,54739	< 0,001
	S22-	S21	2,78796*	,75249	,009
		S22	-4,88706*	,83593	< 0,001
		S22+	-4,41317*	,86242	< 0,001
		S23-	-5,94078*	,81363	< 0,001
		S23	-7,00838*	,80447	< 0,001
	S22	S21	7,67502*	,59266	< 0,001
		S22-	4,88706*	,83593	< 0,001
		S22+	,47389	,72716	,987
		S23-	-1,05372	,66858	,616
		S23	-2,12132*	,65740	,022
	S22+	S21	7,20113*	,62947	< 0,001
		S22-	4,41317*	,86242	< 0,001
		S22	-,47389	,72716	,987
		S23-	-1,52761	,70141	,256
		S23	-2,59522*	,69077	,004
	S23-	S21	8,72874*	,56076	< 0,001
		S22-	5,94078*	,81363	< 0,001
		S22	1,05372	,66858	,616
		S22+	1,52761	,70141	,256
		S23	-1,06760	,62880	,536
	S23	S21	9,79635*	,54739	< 0,001
		S22-	7,00838*	,80447	< 0,001

		S22	2,12132*	,65740	,022
		S22+	2,59522*	,69077	,004
		S23-	1,06760	,62880	,536
**CN6**	S21	S22-	-3,23856*	,85319	,008
		S22	-4,03119*	,81625	< 0,001
		S22+	-3,28415*	,54243	< 0,001
		S23-	-3,58023*	,54487	< 0,001
		S23	-4,37486*	,53778	< 0,001
	S22-	S21	3,23856*	,85319	,008
		S22	-,79262	1,08938	,978
		S22+	-,04558	,90261	1,000
		S23-	-,34167	,90408	,999
		S23	-1,13630	,89982	,803
	S22	S21	4,03119*	,81625	< 0,001
		S22-	,79262	1,08938	,978
		S22+	,74704	,86779	,954
		S23-	,45096	,86932	,995
		S23	-,34368	,86489	,999
	S22+	S21	3,28415*	,54243	< 0,001
		S22-	,04558	,90261	1,000
		S22	-,74704	,86779	,954
		S23-	-,29608	,61941	,997
		S23	-1,09072	,61318	,483
	S23-	S21	3,58023*	,54487	< 0,001
		S22-	,34167	,90408	,999

		S22	-,45096	,86932	,995
		S22+	,29608	,61941	,997
		S23	-,79463	,61534	,789
	S23	S21	4,37486*	,53778	< 0,001
		S22-	1,13630	,89982	,803
		S22	,34368	,86489	,999
		S22+	1,09072	,61318	,483
		S23-	,79463	,61534	,789
**CN7**	S21	S22-	-12,13005*	1,40199	< 0,001
		S22	-18,93698*	1,04489	< 0,001
		S22+	-23,04713*	1,09483	< 0,001
		S23-	-25,19463*	1,15291	< 0,001
		S23	-31,69786*	,99986	< 0,001
	S22-	S21	12,13005*	1,40199	< 0,001
		S22	-6,80694*	1,51191	,001
		S22+	-10,91708*	1,54684	< 0,001
		S23-	-13,06458*	1,58848	< 0,001
		S23	-19,56782*	1,48115	< 0,001
	S22	S21	18,93698*	1,04489	< 0,001
		S22-	6,80694*	1,51191	,001
		S22+	-4,11015*	1,23245	,015
		S23-	-6,25765*	1,28432	< 0,001
		S23	-12,76088*	1,14892	< 0,001
	S22+	S21	23,04713*	1,09483	< 0,001

		S22-	10,91708*	1,54684	< 0,001
		S22	4,11015*	1,23245	,015
		S23-	-2,14750	1,32528	,587
		S23	-8,65074*	1,19452	< 0,001
	S23-	S21	25,19463*	1,15291	< 0,001
		S22-	13,06458*	1,58848	< 0,001
		S22	6,25765*	1,28432	< 0,001
		S22+	2,14750	1,32528	,587
		S23	-6,50324*	1,24797	< 0,001
	S23	S21	31,69786*	,99986	< 0,001
		S22-	19,56782*	1,48115	< 0,001
		S22	12,76088*	1,14892	< 0,001
		S22+	8,65074*	1,19452	< 0,001
		S23-	6,50324*	1,24797	< 0,001
**CN9**	S21	S22-	-10,07440*	1,97187	< 0,001
		S22	-10,25570*	1,29680	< 0,001
		S22+	-6,38081*	1,07598	< 0,001
		S23-	-1,92497	1,04622	,445
		S23	-3,31614*	1,08767	,033
	S22-	S21	10,07440*	1,97187	< 0,001
		S22	-,18130	2,03906	1,000
		S22+	3,69358	1,90625	,400
		S23-	8,14943*	1,88962	,002
		S23	6,75826*	1,91288	,015
	S22	S21	10,25570*	1,29680	< 0,001

		S22-	,18130	2,03906	1,000
		S22+	3,87488*	1,19467	,022
		S23-	8,33073*	1,16794	< 0,001
		S23	6,93956*	1,20521	< 0,001
	S22+	S21	6,38081*	1,07598	< 0,001
		S22-	-3,69358	1,90625	,400
		S22	-3,87488*	1,19467	,022
		S23-	4,45584*	,91658	< 0,001
		S23	3,06468*	,96363	,022
	S23-	S21	1,92497	1,04622	,445
		S22-	-8,14943*	1,88962	,002
		S22	-8,33073*	1,16794	< 0,001
		S22+	-4,45584*	,91658	< 0,001
		S23	-1,39117	,93028	,668
	S23	S21	3,31614*	1,08767	,033
		S22-	-6,75826*	1,91288	,015
		S22	-6,93956*	1,20521	< 0,001
		S22+	-3,06468*	,96363	,022
		S23-	1,39117	,93028	,668
**CN11**	S21	S22-	-5,93315*	1,40052	,002
		S22	-6,63629*	1,38477	< 0,001
		S22+	-3,95715*	1,13566	,009
		S23-	-1,88700	,98749	,401
		S23	-6,17761*	1,12218	< 0,001

	S22-	S21	5,93315*	1,40052	,002
		S22	-,70314	1,72662	,998
		S22+	1,97600	1,53406	,790
		S23-	4,04615	1,42785	,072
		S23	-,24446	1,52411	1,000
	S22	S21	6,63629*	1,38477	< 0,001
		S22-	,70314	1,72662	,998
		S22+	2,67914	1,51970	,496
		S23-	4,74928*	1,41241	,017
		S23	,45868	1,50966	1,000
	S22+	S21	3,95715*	1,13566	,009
		S22-	-1,97600	1,53406	,790
		S22	-2,67914	1,51970	,496
		S23-	2,07015	1,16920	,489
		S23	-2,22046	1,28499	,516
	S23-	S21	1,88700	,98749	,401
		S22-	-4,04615	1,42785	,072
		S22	-4,74928*	1,41241	,017
		S22+	-2,07015	1,16920	,489
		S23	-4,29061*	1,15611	,004
	S23	S21	6,17761*	1,12218	< 0,001
		S22-	,24446	1,52411	1,000
		S22	-,45868	1,50966	1,000
		S22+	2,22046	1,28499	,516
		S23-	4,29061*	1,15611	,004

**CN12**	S21	S22-	4,13398	1,51006	,082
		S22	4,17716*	1,30299	,023
		S22+	2,79131	1,60384	,508
		S23-	-,90559	1,25410	,979
		S23	-1,33705	1,25142	,893
	S22-	S21	-4,13398	1,51006	,082
		S22	,04319	1,32098	1,000
		S22+	-1,34267	1,61850	,961
		S23-	-5,03956*	1,27279	,004
		S23	-5,47103*	1,27015	,001
	S22	S21	-4,17716*	1,30299	,023
		S22-	-,04319	1,32098	1,000
		S22+	-1,38585	1,42725	,926
		S23-	-5,08275*	1,01857	< 0,001
		S23	-5,51422*	1,01527	< 0,001
	S22+	S21	-2,79131	1,60384	,508
		S22-	1,34267	1,61850	,961
		S22	1,38585	1,42725	,926
		S23-	-3,69690	1,38276	,090
		S23	-4,12837*	1,38033	,040
	S23-	S21	,90559	1,25410	,979
		S22-	5,03956*	1,27279	,004
		S22	5,08275*	1,01857	< 0,001
		S22+	3,69690	1,38276	,090

		S23	-,43147	,95172	,998
	S23	S21	1,33705	1,25142	,893
		S22-	5,47103*	1,27015	,001
		S22	5,51422*	1,01527	< 0,001
		S22+	4,12837*	1,38033	,040
		S23-	,43147	,95172	,998

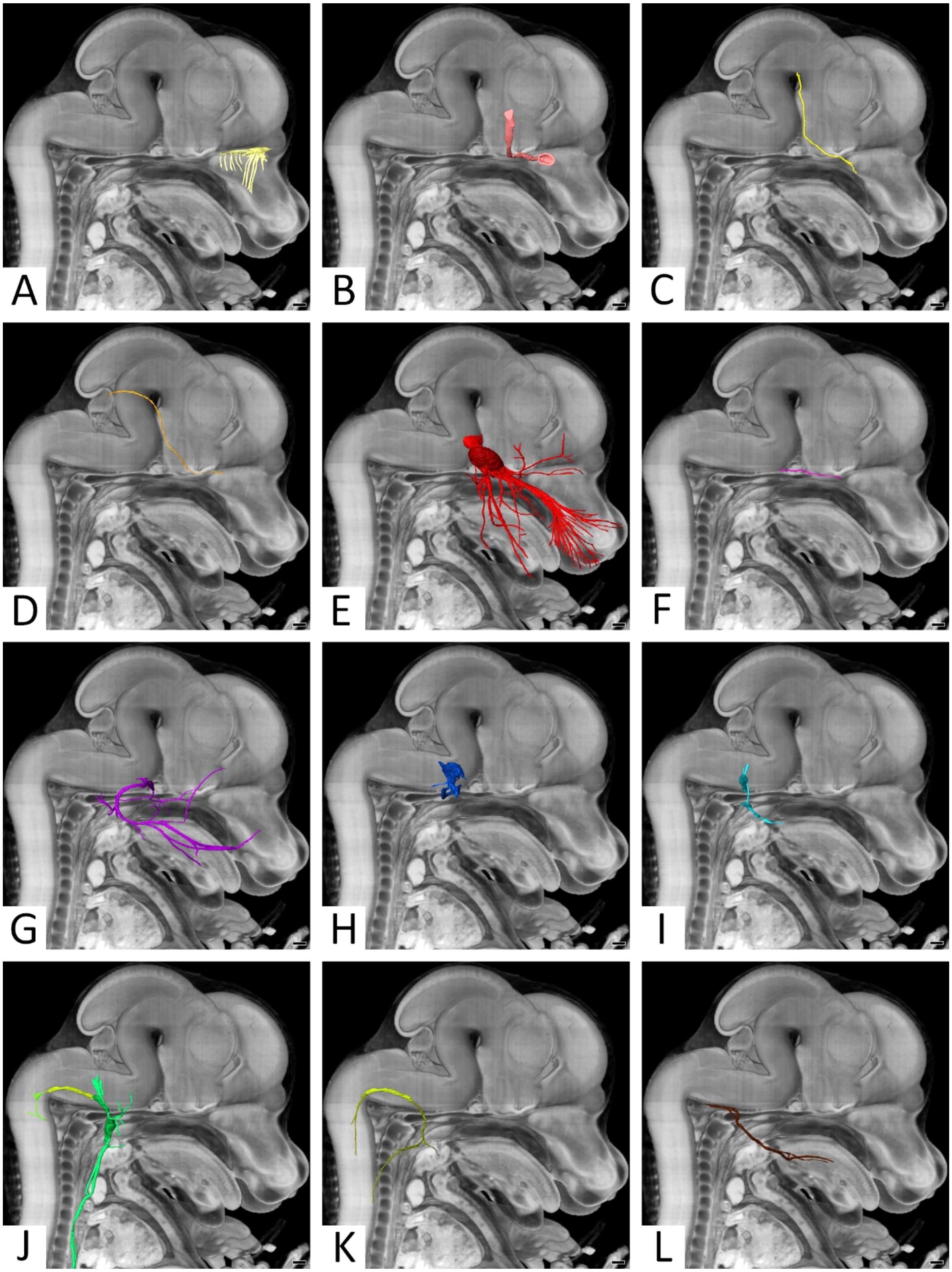

Supplementary Figure 1: Overview of cranial nerves (CN) 1 – 12. **(A-L)** Overview of all right CNs. 3D surface renderings in the context of a volume rendering of a mouse embryo/fetus of S23-. **(A)** CN1 (light yellow), **(B)** CN2 (light red), **(C)** CN3 (yellow), **(D)** CN4 (orange), **(E)** CN5 (red), **(F)**

CN6 (pink), **(G)** CN7 (violet), **(H)** CN8 (blue), **(I)** CN9 (turquoise), **(J)** CN10 (green), **(K)** CN11 (olive), **(L)** CN12 (brown). Scale bars = 250 µm.
